# A general solution for opening double-stranded DNA for isothermal amplification

**DOI:** 10.1038/srep34582

**Published:** 2016-09-30

**Authors:** Gangyi Chen, Juan Dong, Yi Yuan, Na Li, Xin Huang, Xin Cui, Zhuo Tang

**Affiliations:** 1 Natural Products Research Center, Chengdu Institute of Biology, Chinese Academy of Sciences, Chengdu 610041, P. R. China

## Abstract

Nucleic acid amplification is the core technology of molecular biology and genetic engineering. Various isothermal amplification techniques have been developed as alternatives to polymerase chain reaction (**PCR**). However, most of these methods can only detect single stranded nucleic acid. Herein, we put forward a simple solution for opening double-stranded DNA for isothermal detection methods. The strategy employs recombination protein from *E. coli* (**RecA**) to form nucleoprotein complex with single-stranded DNA, which could scan double-stranded template for homologous sites. Then, the nucleoprotein can invade the double-stranded template to form heteroduplex in the presence of ATP, resulting in the strand exchange. The ATP regeneration system could be eliminated by using high concentration of ATP, and the 3′-OH terminal of the invasion strand can be recognized by other DNA modifying enzymes such as DNA polymerase or DNA ligase. Moreover, dATP was found to be a better cofactor for **RecA**, which make the system more compatible to DNA polymerase. The method described here is a general solution to open dsDNA, serving as a platform to develop more isothermal nucleic acids detection methods for real DNA samples based on it.

Highly sensitive and selective DNA detection methods are important scientific tools in molecular biology and medical research. Many analytical methods for specific nucleic acid quantification have been developed^1^,^2^,^3^,^4^,^5^,^6^,^7^. Polymerase chain reaction (**PCR**) is a widely used technology because of its remarkably high rapidity, precision and reproducibility^8^,^9^,^10^,^11^,^12^,^13^,^14^. Based on the thermal cycling system (**PCR** machine), duplex DNA targets and amplicons could be separated at denaturation temperature, yielding two single stranded DNA as templates for next round of amplification. The following primer annealing and elongation on the template will carry out at different temperatures. Therefore, sophisticated equipment for precise temperature control and thermal stable DNA polymerase are indispensable for this method, which has limited its application in unspecialized laboratory and on-site tests. These limitations in PCR-based techniques have spurred the development of a new molecular-biological technique known as isothermal DNA amplification^15^,^16^,^17^,^18^,^19^,^20^,^21^. As only a single optimal reaction temperature is required for the entire detection, isothermal amplification could satisfy rapid on-site detection of environmental, foodborne, and water-borne pathogens as well as for point-of-care clinical diagnostics, providing simpler and more effective reaction conditions without expensive equipment. So far, a lot of isothermal DNA amplification technologies, including **NASBA**^15^, **SDA**^16^, **RCA**^18^, **HDA**^19^, **LAMP**^21^ and so on ref. 22, have been developed as promising alternatives of thermal cycling based technique. However, most of isothermal amplification methods can only detect single-stranded nucleic acid. To apply those methods in the detection of double-stranded DNA, extra steps to obtain the single stranded DNA through heat denaturation are required. One solution to open double stranded DNA is to preheat the DNA sample at 95 °C with two pairs of primers before isothermal amplification (Fig. 1A). Then the outer primers and inner primers are annealed to corresponding unwound single stranded template which would be extended by DNA polymerase with strand displacement activity to displace the elongated product of inner primers, yielding the single-stranded DNA to initiate the following isothermal amplification. However, the pre-denaturation step is dependent of heating equipment, making it impossible for these methods to achieve in one step. Recently, a novel isothermal DNA amplification method named recombinase polymerase amplification (**RPA**) has been reported^20^. Instead of using high temperature to achieve the denaturation of the double-stranded DNA target, **RPA** utilize recombinase enzyme to form complex with ssDNA primer that could search for DNA sequence homology on the double stranded DNA target, facilitating the strand exchange between dsDNA and single stranded primer to form D-loop. Then the primer can be extended by DNA polymerase with strand-displacement activity (Fig. 1B). Compared to other isothermal amplification methods like **HDA**^19^ and **LAMP**^21^ that need preheating to open dsDNA or constant high temperature for entire processes, **RPA** is the only isothermal method that could work at 37 °C because of the application of recombinase. To facilitate the DNA replication, two more proteins besides recombinase **uvsX** are indispensable in **RPA**: **uvsY** for stabilizing interactions between **uvsX** and ssDNA and **gp32** for stabilization of the displaced ssDNA (Fig. 1B), moreover, an ATP regeneration system are required to supplement the ATP consumed in the process of DNA substitution. Three proteins applied in **RPA** are unavailable commercially and all those enzymes are obtained from T4 phage that was a considerable threat for widely used engineered bacterium for large-scale preparation. As the critical component in **RPA**, **uvsX** is the recombinase of T4 phage. Recombinase are ubiquitous proteins found in almost all organisms, including **RecA** from bacteria, **Rad51** and **Dmc1** from eukaryotes, and **RadA** from archaea. They are important proteins participating in many biological processes such as double-strand DNA break (**DSB**) repair, rescue of stalled or collapsed replication forks, chromosomal rearrangements and horizontal gene transfer^23^. Up to now, **RecA** of *E. coli* is the only commercial available recombinase and has been extensively studied. And the substantial function of **RecA** *in vivo* is to form nucleoprotein complex with transient single-stranded DNA and facilitate strand exchange with homologous duplex^24^. Therefore, we ask this question: Can we utilize the recombinase **RecA** of *E. coli* as a tool to open double-stranded DNA for isothermal detection methods. To achieve this goal, there are three challenges we need to address: 1. Even it have been reported that **RecA** can promote the formation of D-loop in the presence of ATP^25^, whether the complex formed by **RecA** and short ssDNA can invade into duplex DNA to substitute the identical strand? 2. It is known that **RecA**, as a single-stranded DNA dependent ATPase, could bind with single-stranded DNA to hydrolyze ATP into ADP immediately and dissociates from single-stranded DNA as a result. However, it is still ambiguous and controversial whether hydrolysis of ATP is needed to realize DNA strand exchange^26^,^27^,^28^,^29^. 3. Though **RecA** could open dsDNA target with short ssDNA, it is not clear whether the 3′-OH terminal of the invasion strand can be recognized by other DNA modifying enzymes such as DNA polymerase or DNA ligase. Herein, we report the research to figure out these problems and establish a general solution to open double-stranded DNA for the different isothermal detection methods using **RecA** protein from *E. coli*.

## Results

### Stoichiometry of binding oligonucleotides by RecA

**Figure 2:**
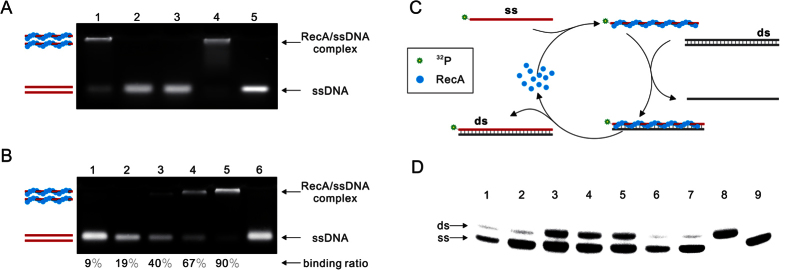
(**A**) 160 pmol **RecA** was incubated with 12 pmol 54 nt oligonucleotides in the presence of different nucleotide cofactor. Lane 1, no nucleotide. Lane 2, ATP. Lane 3, ADP. Lane 4, ATPγS. Lane 5, no **RecA** was added. (**B**) Incremental concentration of **RecA** was incubated with 12 pmol 54 nt oligonucleotides in the presence of ATPγS. Lane 1–6 correspond 20, 40, 80, 120, 160, 0 pmol **RecA** was added respectively. (**C**) Schematic of the process of strand exchange promoted by **RecA**. **RecA** and isotope-labeled ssDNA (**ss**) can form a nucleoprotein complex, then this complex will recognize its homologous double-stranded DNA (**ds**) and displace the identical strand to form a new isotope-labeled heteroduplex. **RecA** will disassemble from the product upon ATP hydrolysis and recycle. (**D**) Result of **RecA** promoted strand exchange between ssDNA oligonucleotides and homologous double-stranded DNA, reaction details could be found in Materials and Methods except the nucleotides cofactor variation: Lane 1, no nucleotides; Lane 2, 1 mM ATP; Lane 3, 1 mM ATP with 10 mM phosphocreatine and 1 U creatine kinase; Lane 4, 5 mM ATP; Lane 5, 5 mM dATP; Lane 6, 5 mM ATPγS; Lane 7, no **RecA**; Lane 8 and 9 are isotope-labeled **ds** and **ss** as markers.

It was reported that **RecA** and ssDNA could form a compressed nucleoprotein complex in the absence of ATP. The complex is inactive for strand exchange, while in the presence of ATP the nucleoprotein will extend to functional structure to perform strand exchange^30^. In addition, there was a constant stoichiometry ratio between **RecA** and nucleotides for forming nucleoprotein. To begin with, we demonstrated the ssDNA binding activity and the stoichiometry of **RecA** either purchased or expressed by ourselves (**Protocol S1**) using gel mobility shift assay^31^. As we can see, **RecA** and ssDNA (54 nt) indeed formed nucleoprotein and generated a low mobility band in the native agarose gel in the absence of ATP (lane 1, Fig. 2A). However, **RecA** disassemble from ssDNA when ATP was added (lane 2, Fig. 2A), which could be caused by ADP produced from hydrolysis of ATP as **RecA**/ssDNA has the strong ATPase activity. This assumption was verified by adding ADP into the reaction, where we found that **RecA** disassemble from ssDNA when ADP was added (lane 3, Fig. 2A). To prevent disassemble of the nucleoprotein we introduced a modified nucleotide adenosine-5′-o-(3-thio-triphosphate) (ATPγS) which could not be hydrolyzed by **RecA**. By incubating various concentration of **RecA** with ssDNA (12 pmol) and then analyzed on native agarose gel, the corresponding binding ratio of ssDNA by **RecA** was obtained. Through calculation, we obtained the average stoichiometry of about 3 nucleotides per **RecA** in the presence of ATPγS (Fig. 2B). In the same way, the stoichiometry of about 5 nucleotides per **RecA** was obtained in the absence of ATP (Figure S1B), consistent with previous research^32^,^33^,^34^,^35^. Therefore, in the following experiment **RecA** was used to completely cover ssDNA based on the 3 nt/**RecA** in the presence of ATP and 5 nt/**RecA** in the absence of ATP.

### RecA promote exchange between oligonucleotides with homologous double-stranded DNA

As nearly all the nucleic acid detection methods depend on hybridization of primer or probe with template, we then check the capability of nucleoprotein complex formed by **RecA** and ssDNA to substitute the identical strand of the homologous double stranded DNA. Here we designed an experiment to demonstrate the strand exchange activity of **RecA**, as shown in Fig. 2C: The single stranded DNA (**ss**) was isotope-labeled, which could form nucleoprotein with **RecA**. If the complex (**RecA**/**ss**) could invade into homologous double stranded DNA (**ds**), the isotope-labeled **ds** should be detected based on the native PAGE analysis. So strand exchange of ssDNA oligonucleotides and their homologous double stranded DNA was tested in our experiment. As illustrated in Figure S2C, no isotope-labeled dsDNA products was detected in the absence of ATP (lane 3, Figure S2C), while the exchanged dsDNA containing isotope-labeled **ss** was observed on the native PAGE in the presence of ATP and the exchange efficiency increased as the ATP concentration increased (lane 4–9, Figure S2C), which verified the DNA strand-substitution catalyzed by **RecA** requires ATP as cofactor. However, the efficiency of strand exchange was very low in the presence of 1 mM ATP, which could be caused by the hydrolysis of ATP by **RecA** in the presence of ssDNA. As **RecA** can form stable nucleoprotein with ssDNA in the presence of ATPγS as shown in the experiment above, we wonder if ATPγS can replace ATP to increase the efficiency. However, when ATPγS was employed, no exchange products were obtained (lane 6, Fig. 2D). Those results implied that ATP hydrolysis was indispensable in the process of strand exchange promoted by **RecA**. Therefore, It was supposed that the low efficiency of strand exchange was caused by hydrolysis of ATP by **RecA**. It is well known that, **RecA** hydrolyze ATP into ADP continuously when it bind with ssDNA, so in all the strand-exchange reaction *in vitro*, an ATP regeneration system (composed of phosphocreatine and creatine kinase) that convert ADP into ATP was included, such as in **RPA**. When ATP regeneration system was introduced into the reaction mixture with 1 mM ATP, the efficiency of strand exchange between ssDNA oligonucleotides and homologous double-stranded DNA increased dramatically (lane 3, Fig. 2D). However, the addition of ATP regeneration system made the reaction more complex and costly. We are very glad to found that this problem could be solved through increasing the concentration of ATP. In the presence of 5 mM ATP, the exchanging efficiency reach to the same level with the reaction containing ATP regeneration system (lane 4 vs. lane 3 in Fig. 2D). It has been reported that dATP is a better cofactor for **RecA**^36^. Therefore, we investigate the feasibility to exchange the ATP in our system with dATP. As illustrated in Fig. 2D, the efficiency of strand exchange catalyzed by **RecA** in the presence of dATP (lane 5) was as same as that in the presence of ATP (lane 4). Besides, the capacity to perform strand exchange of ssDNA of varying length based on this system was investigated, revealing that ssDNA as short as 24 nt was enough for **RecA** promoted strand exchange (Figure S2B). In short, we realized the opening of dsDNA and substitution with a ssDNA by using **RecA** of *E. coli*. And we found that ATP hydrolysis was essential for the whole process of strand exchange. The ATP regeneration system could be eliminated by using high concentration of ATP. Moreover, dATP could be applied as cofactor for **RecA** to perform strand exchange between short ssDNA and homologous double stranded DNA with same efficiency.

### Ligation of two oligonucleotides after strand exchange

**Figure 3:**
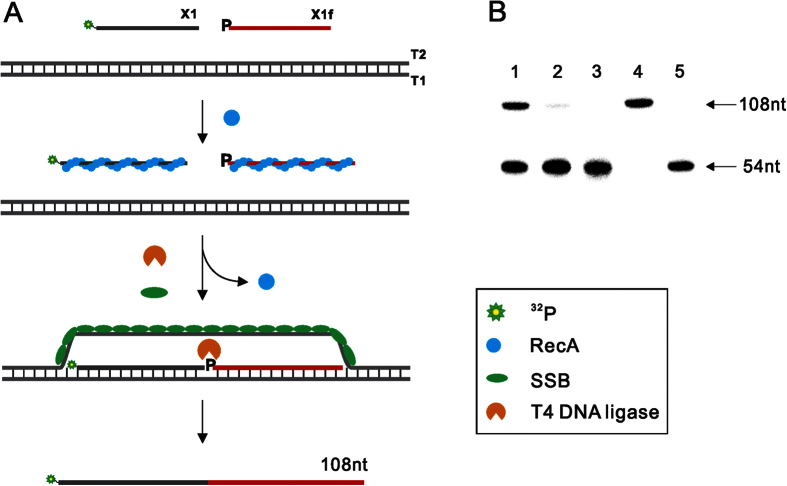
(**A**) Schematic of the process of **RecA** mediated ligation of two ssDNA oligonucleotides using double-stranded DNA as template. Firstly, **RecA** bind with two ssDNA oligonucleotides to form nucleoprotein complex respectively to recognize and unwound double-strand template homologously. Then, the two oligonucleotides annealed with complementary strand in juxtaposition and are ligated by T4 DNA ligase. (**B**) PAGE analysis of **RecA** mediated ligation reaction: Lane 1, the reaction was conducted in presence of dATP. Lane 2, **RecA** was not included. Lane 3, RecA was included while the double-stranded DNA template was removed. Lane 4 and 5, ^32^P-labeled 108 nt and 54 nt oligonucleotide as markers.

As research showed that **RecA** remain associated with the heteroduplex after strand exchange^37^, so after strand exchange, what we care about is whether 3′-OH terminal of the invasion strand can be manipulated by DNA modifying enzyme like DNA ligase. Some isothermal detection methods like **RCA** adopt the strategy of ligation to initiate the nucleic acid detection. Therefore, we designed the experiment to verify the feasibility of ligation reaction of two ssDNA in the presence of duplex template with the aid of **RecA**-based dsDNA opening system. As shown in Fig. 3A, we designed two ssDNA oligonucleotides that were complementary to one strand of the duplex template in juxtaposition. The first one was labeled with isotope and the second one with 5′-phosphate group that is necessary for ligation reaction catalyzed by T4 DNA ligase. Then the nucleoprotein complexes formed by two oligonucleotides with **RecA** could scan double stranded template and invade at the homologous sites to form hetroduplex with a nick in the middle, which would be recognized and ligated by T4 DNA ligase to yield an isotope-labeled product with expected length. However, the ligation efficiency was very low in the presence of high concentration of ATP (lane 1, Figure S4B). We speculated that after nucleoprotein complex invaded into duplex template to form D-loop the displaced strand was likely to re-anneal with the complementary strand and exclude the invasion ssDNA oligonucleotides before they were ligated by T4 DNA ligase. Previous studies reported that single-stranded DNA binding protein (**SSB**) can aid in the formation of nucleoprotein complexes and stimulate DNA strand exchange by binding to the displaced single-stranded DNA^38^,^39^,^40^. Single-strand DNA-binding protein (**SSB**) is a 178-amino-acid-long protein found in *E. coli* that binds to single-stranded regions of DNA. It has important function during all aspects of DNA metabolism: replication, recombination, and repair. As well as stabilizing the single-stranded DNA, **SSB** proteins bind to and modulate the function of numerous proteins involved in all of these processes. Moreover **SSB** protein is easily obtained from commercial sources or direct expression from *E. coli* (Protocol S1 and Figure S3). When **SSB** was introduced into the reaction, the efficiency of ligation increased greatly (lane 2, Figure S4B). Out of expectation, when dATP was employed into the same reaction, the ligation efficiency was further improved (Fig. 3B). As it is well known that ATP is a cofactor of T4 DNA ligase and studies showed that although adenylation enzyme could form in the presence of dATP, it is inactive in the ligation reaction^41^. However, it is surprising to find that dATP could also serve as a cofactor for T4 DNA ligase to work in the condition employed in our experiment (Figure S5). Eventually, we had performed ligation of two oligonucleotides using double-stranded DNA as template at a constant temperature. This would be a great help for the isothermal detection methods which rely on ligation reaction such as rolling circle amplification (**RCA**) to realize the direct detection of double-stranded DNA isothermally.

### Primer extension after strand exchange

**Figure 4:**
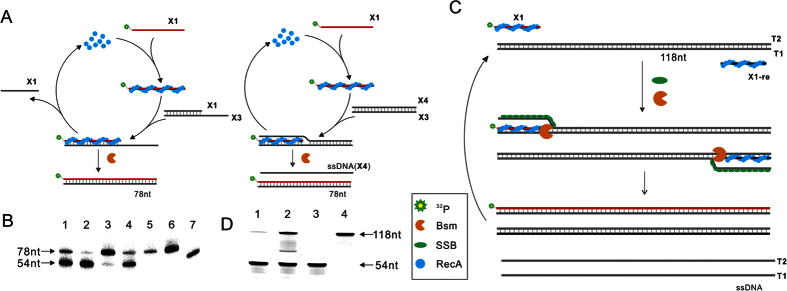
(**A**) Schematic of the process of primer extension after strand exchange on 5′-ssDNA tailed duplex and intact blunt-ended duplex template. Left, after complete strand substitution of the isotope-labeled primer with one strand of the 5′-ssDNA tailed duplex, the isotope-labeled primer was elongated by DNA polymerase. Right, after strand exchange of isotope-labeled primer with one strand of blunt-ended duplex, D-loop formed. Then the isotope-labeled primer was extended by DNA polymerase with strand displacement activity and a single-stranded DNA was released (**B**) Results of primer extension after strand exchange. Lane 1, intact blunt-ended duplex served as template in the presence of **RecA**. Lane 2, intact blunt-ended duplex served as template in the absence of **RecA**. Lane 3, 5′-ssDNA tailed duplex served as template in the presence of **RecA**. Lane 4, 5′-ssDNA tailed duplex served as template in the absence of **RecA**. Lane 5, isotope-labeled primer was extended on the complementary 78 nt single-stranded template to serve as a positive control. Lane 6, ^32^P-labeled 78 nt oligonucleotide. Lane 7, ^32^P-labeled 54 nt oligonucleotide as marker respectively. (**C**) Schematic of **RecA** based DNA amplification. First, single-stranded primers bind with **RecA** to form nucleoprotein complex, which could scan the double-stranded template until the homologous sites are located. Then, following strand exchange, the displaced strand is stabilized by **SSB** and primers are extended by **Bsm** DNA polymerase. After extension, two ssDNA are generated. (**D**) Results of **RecA**-based DNA amplification using blunt-ended duplex as template. Lane 1, in the presence of 5 mM ATP. Lane 2, in the presence of 5 mM dATP. Lane 3 and 4 are 54 nt and 118 nt ^32^P-labeled oligonucleotides as marker respectively.

Besides ligation, most of the isothermal detection methods, such as **SDA** and **LAMP** and so on, rely on generation of single stranded DNA as target, which could be served as template of the primers for DNA polymerase to start the isothermal amplification. So we wonder if the 3′-OH terminal of the invasion strand can be recognized by the DNA polymerase with strand displacement activity, like Bst and Bsm DNA polymerase, to displace one strand of the duplex template after extension. In order to figure out the accessibility of the 3′-OH terminal of the invasion strand by the DNA polymerase, we designed a 5′-ssDNA tailed duplex DNA to serve as template (left, Fig. 4A). In the presence of **RecA** and ATP, almost all the labeled primers were extended to corresponding length (lane 3, Fig. 4B). However, in the absence of **RecA**, only a little background products were observed (lane 4, Fig. 4B). Therefore, it can be concluded that the 3′-OH terminal of the primer was accessible for DNA polymerase to elongate after strand exchange. However, the natural DNAs are always intact duplex, unlike the case of 5′-ssDNA tailed duplex DNA where the displaced strand was completely stripped off the heteroduplex. As shown in Fig. 4A (right), after **RecA** assisted the ssDNA oligonucleotide to invade into intact duplex to form D-loop, the displaced strand was not completely dissociated and the downstream still paired^42^,^43^,^44^,^45^. This structure was not stable because the primer may be excluded through re-annealing of the displaced strand as referred above. So we designed an blunt-ended duplex as a target to explore if the polymerase could recognize at the 3′-OH terminal and overcome the barrier of downstream base-pair through displacing it (right, Fig. 4A). In the presence of **RecA**, almost half of the labeled primers were extended to corresponding length (lane 1, Fig. 4B), while in the absence of **RecA** only a little products were obtained as background (lane 2, Fig. 4B). The results revealed that isotope-labeled primer have invaded into double-stranded template, and it could be extended by the DNA polymerase with strand displacement activity to release a ssDNA. Although the efficiency of extension using blunt-ended duplex as template was lower than that of 5′-ssDNA tailed duplex DNA, it can be concluded that strand exchange promoted by **RecA** is compatible to DNA polymerase with strand displacement activity and ssDNA will be released in the process of DNA replication.

### The RecA based DNA amplification

We have obtained ssDNA through **RecA** promoted strand exchange followed by extension of the invading strand with DNA polymerase. However, the yield was not very high (lane 1, Fig. 4B). So we wonder if the displaced ssDNA can serve as template for another ssDNA primer, the amplification of templates should carry on (Fig. 4C). As a result, more ssDNA for isothermal amplification template could be produced in the process of amplification, which would be a great advantage for isothermal detection methods. However, no amplification products were obtained in the presence of ATP (lane 2, Figure S6B). We deduced that when two primers were used, a lot of ssDNA that were complementary to each other were generated. As mentioned before, **SSB** can be used to bind with these ssDNA which could serve as template for the primers before they annealed to each other. Consistent with our expectations, amplification products were obtained when **SSB** was employed (lane 1, Figure S6B). However, the efficiency of amplification was still not very high, we speculated that **RecA** remain associated with the heteroduplex after strand exchange^37^ and the homologous sites could be open by nucleoprotein complex only when **RecA** dissociated from the heteroduplex by hydrolysis of ATP. This may be improved by adding dATP, which was hydrolyzed faster than ATP^36^. It was very glad to find that in the presence of dATP the amplification efficiency greatly increased (lane 2, Figure 4D). Besides, we demonstrated oligonucleotides with 54 nt or 35 nt in length could all be used as primers for amplification of blunt-ended duplex(Figure S6C). In addition to blunt-ended duplex, plasmid DNA could also serve as template for **RecA** based DNA amplification, and the amplification products were also visible on stained native agarose gel (Figure S7). Through analysis on multifunctional laser scanning imaging system, we calculated that the templates were amplified 240 times, which implies that more than 240 fold ssDNA were produced in the process of amplification. Although previous research have established an amplification method which can target double-stranded DNA isothermally by introducing recombinase from T4 phage^20^. However, as mentioned before, the proteins they used were all from T4 phage and unavailable commercially. Besides, recombinase would continuously hydrolyze ATP, so an ATP regeneration system should be included. Here, by using commercially available recombinase of *E. coli* (**RecA**), we realized the multiplex ssDNA templates could be produced and have eliminated the need for ATP regeneration system by introducing dATP.

## Discussion

In summary, we have put forward a simple solution for opening double-stranded DNA for isothermal detection methods through invasion of ssDNA into double-stranded DNA in assist of **RecA** protein of *E. coli*. After the ssDNA annealed with complementary strand of the double-stranded template, it can be manipulated by other DNA modified enzyme like DNA ligase and DNA polymerase. The **RecA**-based dsDNA opening system described here appears to have several promising features for research and diagnostic applications as follows: All proteins (**RecA** and **SSB**) used in our method are commercially available, and they can be expressed easily in ordinary laboratory for large-scale preparation; **RecA** has been reported to promote strand exchange reaction with a high sequence specificity^46^, so once combined with other isothermal amplification strategies it could improve the specificity of whole method; The ATP regeneration system is eliminated in our system, which make this method simple and cost-efficient. Moreover, dATP could serve as the cofactor of **RecA** to perform strand exchange in place of ATP, offering more flexibility to our system; After the strand exchange, the 3′-OH terminal of the invasion strand was proved to be accessible to other DNA modifying enzymes like DNA ligase or polymerase, which is compatible with almost all reported isothermal detection methods. By designing a padlock probe with two binding arms complementary to one strand of double stranded template in juxtaposition, rolling circle amplification (**RCA**) can detect dsDNA^18^. Based on our solution, starting structure of loop-mediated isothermal amplification (**LAMP**) can produce at a constant temperature^21^ and the double-stranded DNA with Nickase site at both ends are obtained using only one pair of primers at a constant temperature^16^. By introducing T7 promoter sequence on the 5′ terminal of primers, Nucleic Acid Sequence Based Amplification (**NASBA**) can conduct using dsDNA as substrate^15^. Therefore, The **RecA**-based dsDNA opening system described here is a general solution to open dsDNA, serving as a platform to develop more isothermal nucleic acids detection methods for real DNA samples.

## Materials and Methods

### Oligonucleotides, enzymes, and other reagents

All oligonucleotides were purchased from Sangon Biotech (Shanghai, China) and are shown in Table S1. Bsm DNA Polymerase (Large Fragment), T4 polynucleotide kinase were purchased from Thermo Scientific. Plasmid pET-28(a) was purchased from Novagen, now part of Merck Biosciences. Trans5α Chemically Competent Cell, BL21(DE3) Chemically Competent Cell, FastPfu DNA Polymerase and dNTP were purchased from TransGen Biotech (Beijing, China). Ni–agarose His tag protein purification kit was bought from Beijing CoWin Biotech. [γ-^32^P]ATP was purchased from Furui Biological Engineering (Beijing, China).

### Labelling Reaction

A reaction mixture containing oligonucleotides X1 and X1-35 with 50 mM Tris-HCl (pH 7.8), 40 mM NaCl, 10 mM MgCl_2_, 1 mg/mL BSA, 10 μCi [γ-^32^P]ATP and 10 units of Polynucleotide kinase (PNK) was incubated for 1 h at 37 °C for DNA phosphorylation. The labelled product was purified by 10% denaturing polyacrylamide gel.

### DNA binding assays

Reactions were performed by incubating indicated concentration of purified proteins with 12 pmol X1 (Table S1) at 37 °C for 10 min. Standard conditions were 25 mM Tris (pH 7.6), 1 mM Magnesium chloride and 1 mM DTT. The reaction volume of all binding assays was 10 μl. After the reaction, 2.5 ul 6* loading buffer (0.25% bromophenol blue, 36% sucrose and 30 mM EDTA) was added into each tube. Subsequently, all the samples were loaded on 2% native agarose gel. The results were analyzed by Typhoon FLA 7000 IP and the stoichiometry of **RecA** to oligonucleotides was determined through calculation (GE Healthcare).

### DNA exchange assays

Incubate 0.05 μM ^32^P-labelled oligonucleotides X1 with 18 pmol **RecA** that completely cover the ssDNA oligonucleotides based on the stoichiometry in the standard condition as follows: 25 mM Tris (pH 7.6), 10 mM Magnesium chloride, 1 mM DTT, nucleotide cofactors used were as shown in the Figure. Then 0.05 μM homologous double-stranded DNA formed by annealing of the same unlabeled oligonucleotides X1 with their complementary strand X1C (Table S1) was added. When ATP regeneration system was included, phosphocreatine was 15 mM, creatine kinase was 1 U. The reaction volume was 10 μl. The incubation last for 10 min at 37 °C. After the reaction, the mixture was extracted by phenol-chloroform and precipitated with ethanol. The sediment was dried by Vacuum Drier and loaded on 12% native polyacrylamide gel. The results were analyzed by Typhoon FLA 7000 IP (GE Healthcare).

### Ligation of two oligonucleotides after strand exchange

Incubate 0.05 μM ^32^P-labelled X1 and 0.05 μM 5′-phosphorylated X1f with 18 pmol **RecA** in condition as follows: 25 mM Tris (pH 7.6), 10 mM Magnesium chloride, 1 mM DTT, nucleotide cofactors used were as shown in the Figure. Then 0.05 μM double-stranded DNA(formed by annealing of T1 and T2), 15 pmol **SSB**, 5 U T4 DNA ligase was added followed by incubating for 1 h. After that, 1% SDS and 1 U proteinase K were added and incubated for another 15 min. Afterwards, the reactions were extracted by phenol-chloroform and precipitated with ethanol. The sediment was dried by Vacuum Drier and loaded on 10% denaturing polyacrylamide gel. The results were analyzed by Typhoon FLA 7000 IP(GE Healthcare).

### Extension after DNA exchange

We designed a 5′-ssDNA tailed duplex DNA which was formed by unlabeled X1 annealed with X3 and a blunt-ended duplex DNA annealed with X3 and X4 (Table S1). First, we demonstrated ^32^P-labelled X1 can substitute the unlabeled X1 in the 5′-ssDNA tailed duplex DNA and extended by DNA polymerase. The reaction condition were as follows: 25 mM Tris(PH 7.6), 10 mM Magnesium chloride, 5 mM ATP, 0.25 mM dNTP, 210 pmol **RecA**, 4 U Bsm, 1 mM DTT. Primer and template used in this reaction were ^32^P-labelled X1 0.05 μM, 5′-ssDNA tailed duplex DNA 0.05 μM. Then, we demonstrated ^32^P-labelled X1 can exchange with the blunt-ended duplex and extended by DNA polymerase with strand displacement activity. The reaction condition were as follows: 25 mM Tris(PH 7.6), 10 mM Magnesium chloride, 5 mM ATP, 0.25 mM dNTP, 210 pmol **RecA**, 4 U Bsm, 1 mM DTT. Primer and template used in this reaction was ^32^P-labelled X1 0.05 μM, blunt-ended duplex 0.05 μM. After incubation at 37 °C for 1 h, the reactions were extracted by phenol-chloroform and precipitated with ethanol. The sediment was dried by Vacuum Drier and loaded on 10% denaturing polyacrylamide gel. The results were analyzed by Typhoon FLA 7000 IP(GE Healthcare).

### Amplification using two primers

Pre-incubation of the mixture of 25 mM Tris(pH 7.6), 8% (m/v)PVP(Polyvinyl pyrrolidone), 6 mM Magnesium chloride, 1 mM DTT, 420 pmol **RecA**, 0.3 μM forward primer X1, 0.3 μM reverse primer X1-re and 5 mM nucleotide cofactors shown in the Figure for 5 min at 37 °C. Subsequently the mixture of double-stranded template formed by annealing of T1 and T2 (blunt-ended duplex), 250 μM dNTP, 360 pmol **SSB** and 4 U Bsm was added, the final concentration of template was 625 pM. The incubation continued at 37 °C for 1 h and the reaction was extracted by phenol-chloroform and precipitated with ethanol. The sediment was dried by Vacuum Drier and loaded on 10% denaturing polyacrylamide gel or 4% agarose gel. The results were analyzed by Typhoon FLA 7000 IP(GE Healthcare).

## Additional Information

**How to cite this article**: Chen, G. *et al.* A general solution for opening double-stranded DNA for isothermal amplification. *Sci. Rep.* **6**, 34582; doi: 10.1038/srep34582 (2016).

## Supplementary Material

Supplementary Information

## Figures and Tables

**Figure 1:**
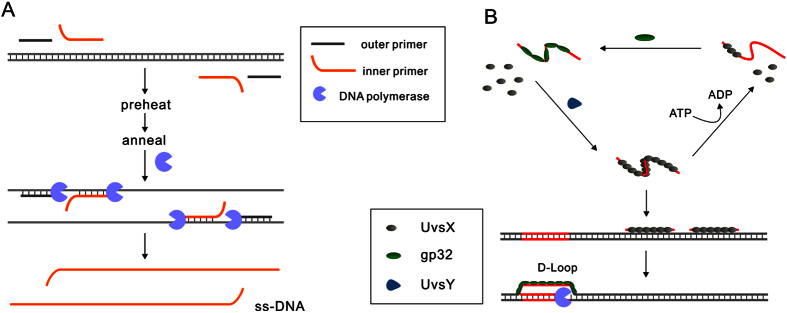
(**A**) The strategy to open dsDNA to produce ssDNA for isothermal detection methods. After the double-stranded DNA is denatured by heating, two pairs of ssDNA primer could anneal to the corresponding single-stranded DNA. Then the primers are extended by DNA polymerase with strand displacement activity. The extension of the outer primers results in the displacement of the elongated products of the inner primers, forming ssDNA. (**B**) Dynamic processes in recombinase polymerase amplification (**RPA**). In the presence of ATP, **uvsX** bind with oligonucleotides cooperatively, upon ATP hydrolysis, the nucleoprotein disassembles and **uvsX** is displaced by **gp32**, but **uvsY** can assist **uvsX** rebind with oligonucleotides. The stable nucleoprotein search on the double-stranded DNA and promote strand exchange at homologous sites with the displaced strand that will be stabilized by **gp32**. Then the oligonucleotides are extended by DNA polymerase with strand displacement activity and a single-stranded DNA was released.
