# Supplementary material for: A general solution for opening double-stranded DNA for isothermal amplification
Source: Sci Rep. 2016 Sep 30;6:34582. doi: 10.1038/srep34582 (PMC5043356; doi:10.1038/srep34582)
Supplement: Supplementary Information [file srep34582-s1.pdf]

Supplementary Information

A general solution for opening double-stranded DNA for isothermal amplification

Gangyi Chen, Juan Dong, Yi Yuan, Na Li, Xin Huang, Xin Cui* and Zhuo Tang*

Supplementary Materials and Methods

Table S1. oligonucleotides used in the experiment

T1(118 nt)	GAATTCTAATACGATGCACTATAGGGACAATTACTATTTACAATTACAATGGACTACAA GATGATTTAGCTCCTAGACCTTTAGCAGCAAGGTCCATATCTGACTTTTTGTTAACGT A
T2(118 nt)	TACGTTAACAAAAAGTCAGATATGGACCTTGCTGCTAAAGGTCTAGGAGCTAAATCAT CTTGTAGTCCATTGTAATTGTAAATAGTAATTGTCCCTATAGTGCATCGTATTAGAATTC
X1(54 nt)	5'-TACGTTAACAAAAAGTCAGATATGGACCTTGCTGCTAAAGGTCTAGGAGCTAAA-3'
X1f(54 nt)	TCA TCT TGT AGT CCA TTG TAA TTG TAA ATA GTA ATT GTC CCT ATA GTG AGT CGT
S1	TGG ACT ACA AGA TGA TTT AGC TCC TAG ACC
X1C(54 nt)	TTT AGC TCC TAG ACC TTT AGC AGC AAG GTC CAT ATC TGA CTT TTT GTT AAC GTA
X1-re(54 nt)	GAATTCTAATACGATGCACTATAGGGACAATTACTATTTACAATTACAATGGAC
X1-35(35 nt)	TACGTTAACAAAAAGTCAGATATGGACCTTGCTGC
X1-re35(35 nt)	GAATTCTAATACGATGCACTATAGGGACAATTACT
X3(78 nt)	AGA TGA GGC ATA GCA GCA GGA TGA TTT AGC TCC TAG ACC TTT AGC AGC AAG GTC CAT ATC TGA CTT TTT GTT AAC GTA
X4(78 nt)	TAC GTT AAC AAA AAG TCA GAT ATG GAC CTT GCT GCT AAA GGT CTA GGA GCT AAA TCA TCC TGC TGC TAT GCC TCA TCT
X1-24(24 nt)	TAC GTT AAC AAA AAG TCA GAT ATG
X1-24C(24 nt)	CAT ATC TGA CTT TTT GTT AAC GTA
X1-32(32 nt)	TAC GTT AAC AAA AAG TCA GAT ATG GAC CTT GC
X1-32C(32 nt)	GCA AGG TCC ATA TCT GAC TTT TTG TTA ACG TA
X1-40(40 nt)	TAC GTT AAC AAA AAG TCA GAT ATG GAC CTT GCT GCT AAA G
X1-40C(40 nt)	CTT TAG CAG CAA GGT CCA TAT CTG ACT TTT TGT TAA CGT A
X1-48(48 nt)	TAC GTT AAC AAA AAG TCA GAT ATG GAC CTT GCT GCT AAA GGT CTA GGA
X1-48C(48 nt)	TCC TAG ACC TTT AGC AGC AAG GTC CAT ATC TGA CTT TTT GTT AAC GTA

Protocol S1. Protein Expression and Purification

The recA and ssb gene was amplified from Escherichia coli strain BJ5183 and cloned into pET28a to form recombinant plasmid pET28a-recA and pET28a-ssb, respectively. The recombinant plasmids were transformed into BL21(DE3) Chemically Competent Cell. Clones were inoculated into 5 ml LB medium and grew overnight, then expand to 400 ml LB medium and cultivated until OD600 was about 0.6. Proteins were expressed by inducing with 1 mM IPTG for 24 h at 16 ℃. After centrifugation and resuspended in starting buffer(20 mM Tris–HCl (pH 8.0), 500 mM NaCl), lysozyme and PMSF was added to 1 mg/ml and 1 mM respectively and then incubated on ice for 1 h followed by centrifugation at 4 ℃. The supernatant was filtered through 0.45 μm millipore filter and purified using Ni–agarose His tag protein purification kit according to the instruction. The purified protein was analyzed by SDS-PAGE Assay.

Protocol S2. Preparation of plasmid DNA for template

Mix equivalent ssDNA T1 and T2 with the final concentration of 1 μM using deionized water. Then heat this mixture to 94 ℃ followed by cooling to room temperature. Add 5 μl this mixture into microcentrifuge tube with 1 μl 10*Taq buffer(200 mM Tris-HCl pH 8.4, 200 mM KCl, 100 mM $(NH_4)_2SO_4$, 200 mM $MgSO_4$), 1 μl dNTP(10 mM each), 2.5 U Taq DNA polymerase, add deionized water to 10 μl, then incubate this mixture at 72 ℃ for 5 min. After that, 4 μl of the reaction was mixed with 1 μl pEASY-T1 Cloning Vector(from TransGen Biotech) and incubate for 10 min at 25 ℃ for ligation. The ligation products were transformed into Trans1-T1 Chemically Competent Cell. Recombinant clones were certified through sequencing and inoculated into liquid LB to cultivate overnight. The recombinant plasmids(named by T1-ds) were extracted using Plasmid Miniprep Kit(from Axgen) and the concentration was determined using ultraviolet spectrophotometer(from Mapada Instrument co., LTD).

Supplementary Figures

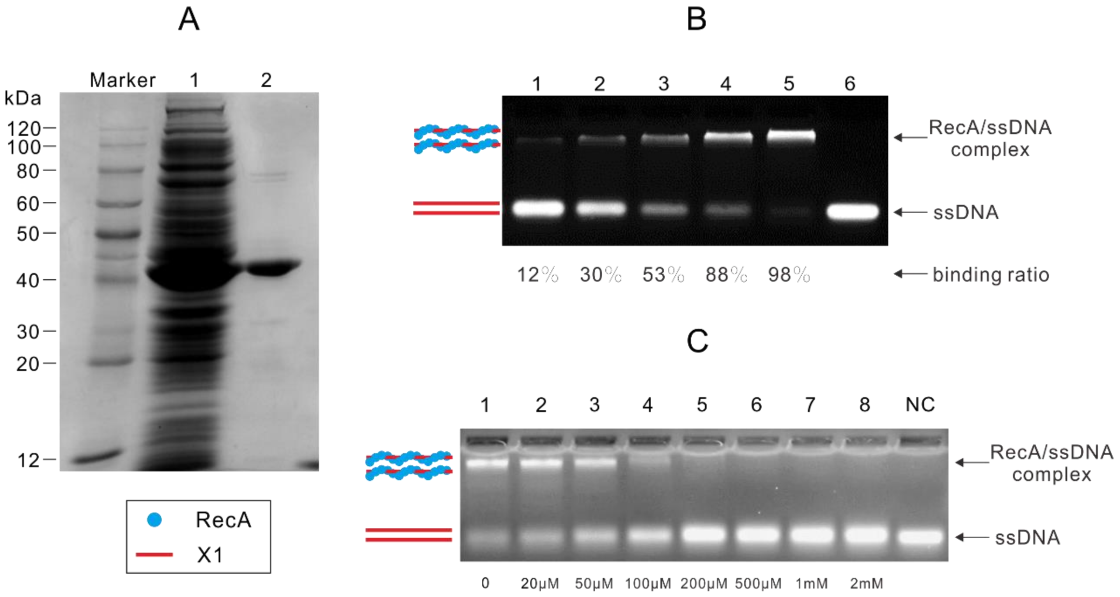

Figure S1. (A) **Expression of RecA of *E. coli* and analyzed on SDS-PAGE gel.** After induced by 1 mM IPTG, 1.2 L bacterial culture was centrifuged and resuspended in 64 ml starting buffer. Then the bacterial culture was lysed by lysozyme followed by centrifugation at 4 ℃. Lane 1, supernatant of the lysate. Lane 2, purification product of the supernatant by nickel column chromatography. Running position of molecular weight markers is indicted on the left. (B) **Analysis of Stoichiometry of RecA to nucleotide in the absence of ATP.** Incremental concentration of purified **RecA** incubated with 12 pmol oligonucleotides X1 in the condition: 25 mM Tris (pH 7.6), 10 mM Magnesium chloride, 1 mM DTT at 37 ℃ for 10 min. Then 2.5 μl 6* loading buffer(0.25% bromophenol blue, 36% sucrose and 30 mM EDTA) was added and all the samples were loaded on 2% native agarose gel. Through analysis on multifunctional laser scanning imaging system, the corresponding binding ratio of different **RecA** concentration was obtained. Then the average stoichiometry of **RecA** to nucleotide was calculated. Lane 1-6 correspond 20,40,80,120,160,0 pmol **RecA** was added respectively. (C) **RecA bind with ssDNA at various concentration of ATP.** 160 pmol **RecA** was incubated with 12 pmol oligonucleotides(54 nt) in the presence of varying concentration of ATP as annotated at the bottom of each lane. NC represent **RecA** is not added.

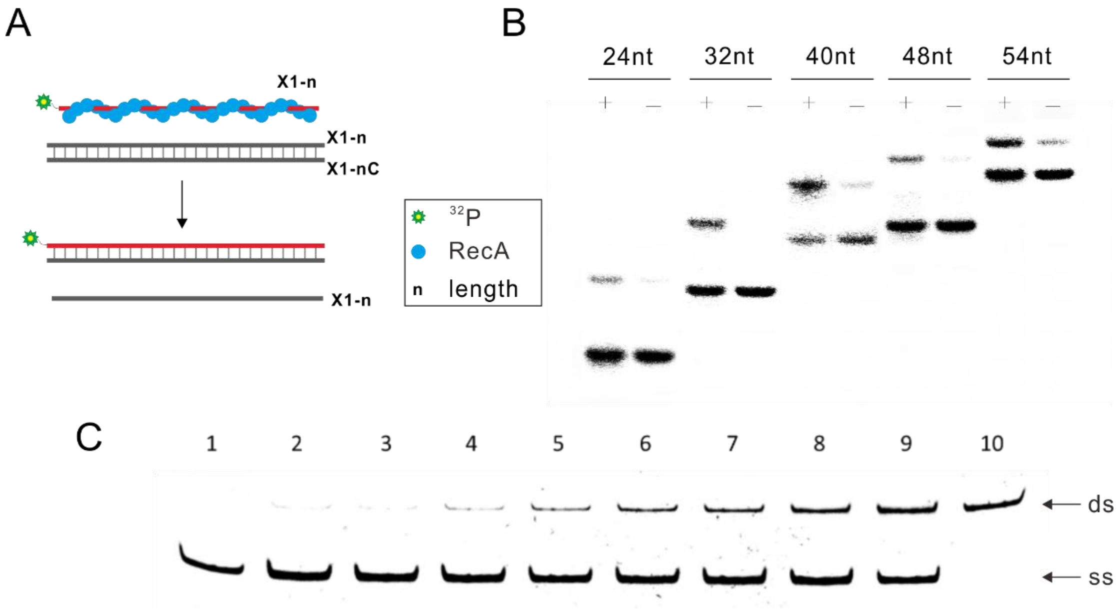

Figure S2. (A and B) **RecA promoted strand exchange of oligonucleotides of varying length (24 nt, 32 nt, 40 nt, 48 nt, 54 nt) with their homologous double-stranded DNA. RecA** was incubated with ^{32}P labelled oligonucleotides with different length(X1-24, X1-32, X1-40, X1-48, X1) as annotate on the figure in the condition: 25 mM Tris (pH 7.6), 10 mM Magnesium chloride, 5 mM ATP, 1 mM DTT at 37 ℃ for 5 min and then homologous double stranded DNA(formed by annealing of unlabeled oligonucleotides with its complementary strand, e.g. X1-24 annealed with X1-24C) was added. The reaction mixture was incubated for another 10 min. After the reaction, the mixture was extracted by phenol-chloroform and precipitated with ethanol. The sediment was dried by Vacuum Drier and loaded on 12% native polyacrylamide gel. The results were analyzed by Typhoon FLA 7000 IP(GE Healthcare). "+" represent **RecA** was added, "-" represent **RecA** wasn't added. (C) **strand exchange between ssDNA oligonucleotides and homologous double-stranded DNA in the presence of various concentration of ATP.** Lane 1, isotope-labeled **ss** as markers; Lane 2, in the absence of **RecA**; Lane 3, in the absence of ATP; Lane 4, 0.5 mM ATP; Lane 5, 1 mM ATP; Lane 6, 2 mM ATP; Lane 7, 3 mM ATP; Lane 8, 4 mM ATP; Lane 9, 5 mM ATP; Lane 10, isotope-labeled **ds** as markers.

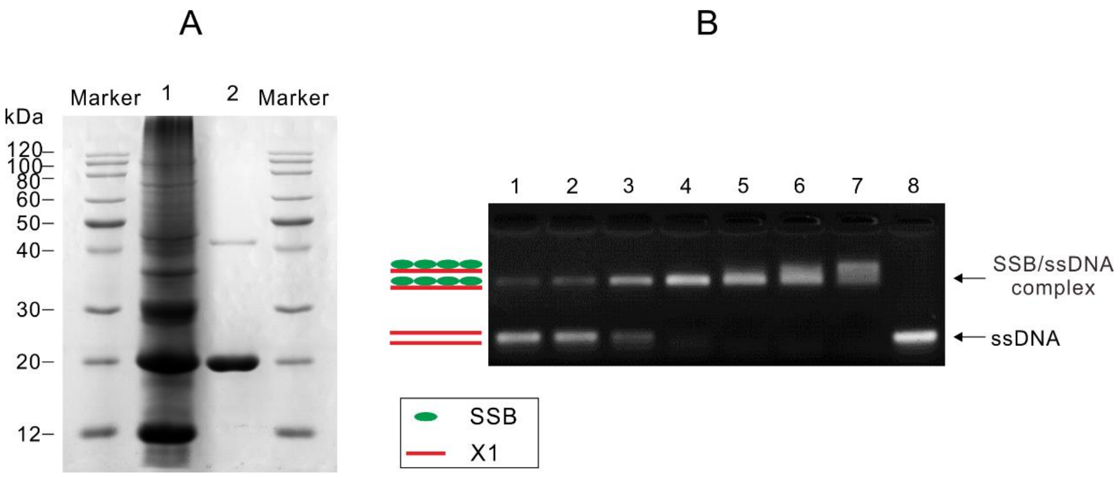

Figure S3. Expression of SSB of *E. coli* and verification of its activity of binding ssDNA oligonucleotides. (A) After induced by 1 mM IPTG, 1.2 L bacterial culture was centrifuged and resuspended in 64 ml starting buffer(20 mM Tris-HCl, PH 8.0, 500 mM NaCl). Then the bacterial culture was lysed by lysozyme followed by centrifugation at 4 ℃. The supernatant and purified product by nickel column chromatography were run on 12% SDS-PAGE gel. Lane 1, supernatant of the lysate. Lane 2, purified product of the supernatant by nickel column chromatography. Running position of molecular weight markers is indicted on the left. (B) Incremental concentration of purified **SSB** incubated with 12 pmol oligonucleotides X1 in the condition: 25 mM Tris (pH 7.6), 10 mM Magnesium chloride, 1 mM DTT at 37 ℃ for 10 min. Then 2.5 μl 6* loading buffer(0.25% bromophenol blue, 36% sucrose and 30 mM EDTA) was added and all the samples were loaded on 2% native agarose gel. The results were analyzed by Typhoon FLA 7000 IP. Lane 1 to 7 correspond to 10, 20, 40, 80, 160, 320, 640, 0 pmol **SSB** protein respectively.

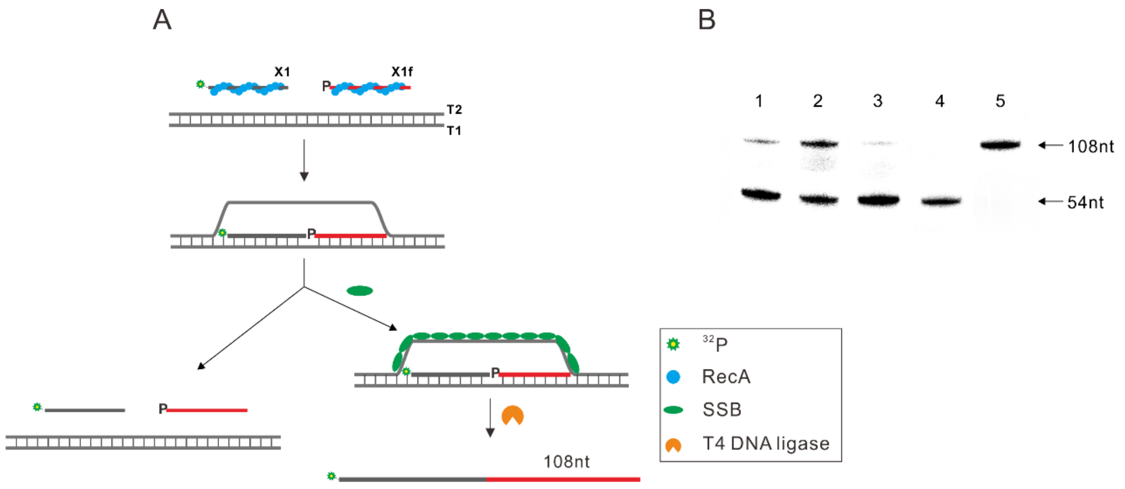

Figure S4. The necessity of SSB in reaction of RecA promoted ligation of two 54 nt oligonucleotides on double-stranded DNA template. Incubate 18 pmol **RecA** with 0.05 μM ^{32}P-labelled **X1** and 0.05 μM 5'-phosphorylated **X1f** in the condition: 25 mM Tris (pH 7.6), 10 mM Magnesium chloride, 5 mM ATP, 1 mM DTT for 5 min. Then 0.05 μM double-stranded DNA template(formed by annealing of T1 and T2), 5 U T4 DNA ligase and 15 pmol **SSB** if present was added followed by incubating for 1 h. After that, 1% SDS and 1 U proteinase K were added and incubated for another 15 min. Afterwards, the reactions were extracted by phenol-chloroform and precipitated with ethanol. The sediment was dried by Vacuum Drier and loaded on 10% denaturing polyacrylamide gel. The results were analyzed by Typhoon FLA 7000 IP(GE Healthcare). Lane 1, ATP only; Lane 2, ATP and **SSB**; Lane 3, **RecA** was not added to serve as negative control; Lane 4 and 5 are ^{32}P-labelled 54 nt and 108 nt oligonucleotides as marker respectively.

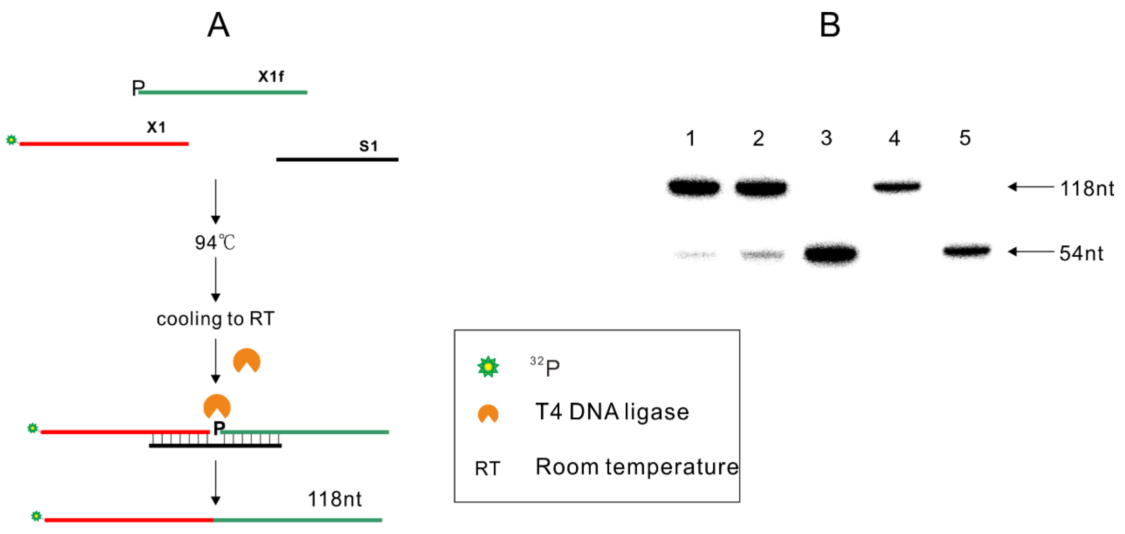

Figure S5. dATP could serve as cofactor of T4 DNA ligase. Ligation of two 54 nt oligonucleotides was conducted in the presence of a short splint as shown in **A**. The first oligonucleotide(X1) was isotope labelled and the second one(X1f) with 5'-phosphate group. Lane 1 was conducted in the presence of ATP, lane 2 was conducted in the presence of dATP, no nucleotide was included in lane 3, lane 4 and 5 were oligonucleotides with 108 nt and 54 nt in length as marker respectively.

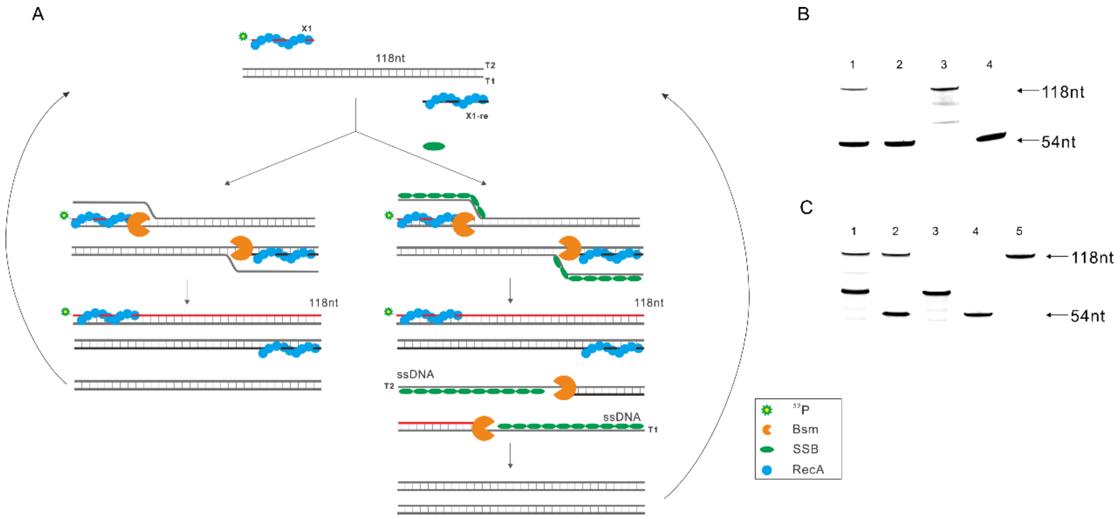

Figure S6. SSB is necessary for RecA based DNA amplification and 54 nt and 35 nt oligonucleotides pairs could both serve as primers for RecA based DNA amplification. 420 pmol **RecA** and 0.3 μM isotope-labelled forward primer X1, 0.3 μM reverse primer X1-re were incubated in the condition: 25 mM Tris(pH 7.6), 8%(m/v)PVP(Polyvinyl pyrrolidone), 6 mM Magnesium chloride, 5 mM ATP, 1 mM DTT at 37 °C for 5 min. Then the mixture of 625 pM double-stranded template(formed by annealing of T1 and T2), 250 μM dNTP, 360 pmol **SSB** and 4 U Bsm was added. The incubation continued at 37 °C for 1 h and the reaction was extracted by phenol-chloroform and precipitated with ethanol. The sediment was dried by Vacuum Drier and loaded on 10% denaturing polyacrylamide gel. The results were analyzed by Typhoon FLA 7000 IP(GE Healthcare). (B) Lane 1, **SSB** was included. Lane 2, **SSB** was not included. Lane 3 and 4 are 54 nt and 118 nt oligonucleotides as marker respectively. (C) Reaction was conduct as in **B** in the presence of SSB except that ATP was replaced by dATP. Lane 1, 54 nt oligonucleotides X1 and X1-re served as primer. Lane 2, 35 nt oligonucleotides X1-35 and X1-re35 served as primer. Lane 3 and 4, Reaction of lane 1 and 2 in the absence of **RecA** to serve as negative control. Lane 5, 118 nt oligonucleotides as amplified product marker.

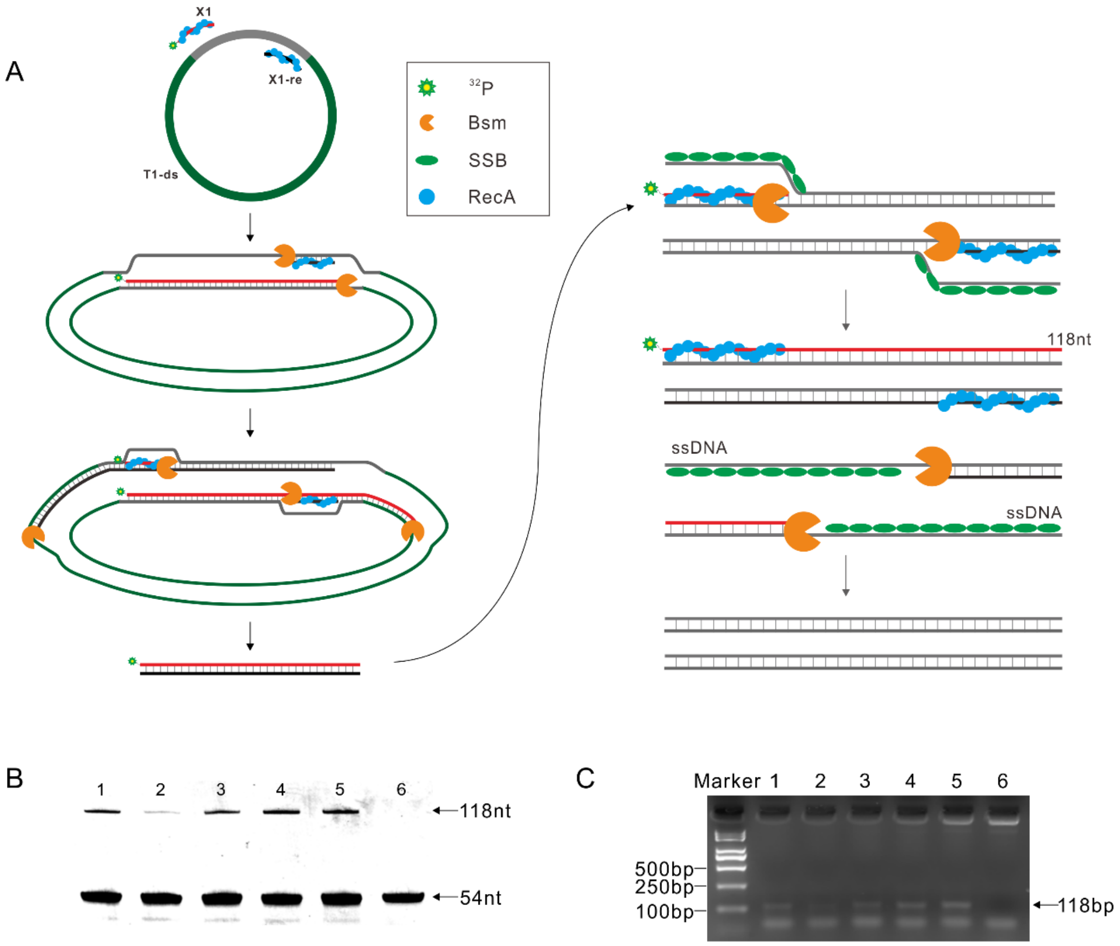

Figure S7. Plasmid DNA could serve as template for RecA-based DNA amplification. 420 pmol **RecA** and 0.3 μM isotope-labelled forward primer X1, 0.3 μM reverse primer X1-re were incubated in the condition: 25 mM Tris(pH7.6), 8%(m/v)PVP(Polyvinyl pyrrolidone), 6 mM Magnesium chloride, 5 mM dATP, 1 mM DTT at 37 °C for 5 min. Then the mixture of 625 pM blunt-ended template(formed by annealing of T1 and T2) and incremental concentration of plasmid DNA T1-ds(see **Protocol S2**), 250 μM dNTP, 360 pmol **SSB** and 4 U **Bsm** was added. The incubation continued at 37 °C for 1 h and the reaction was extracted by phenol-chloroform and precipitated with ethanol. The sediment was dried by Vacuum Drier and half was loaded on 10% denaturing polyacrylamide gel, the other half was loaded on 4% native agarose gel. The results were analyzed by Typhoon FLA 7000 IP(GE Healthcare). (B) Lane 1, 625 pM blunt-ended template(formed by annealing of T1 and T2). Lane 2-6, 562.5 pM, 1.125 nM, 2.25 nM, 4.5 nM, 0 nM T1-ds respectively, (C)Half of the volume of reaction in (B) was analyzed by 4% native agarose gel.
